# Demographic responses to climate change in a threatened Arctic species

**DOI:** 10.1002/ece3.7873

**Published:** 2021-07-14

**Authors:** Kylee D. Dunham, Anna M. Tucker, David N. Koons, Asheber Abebe, F. Stephen Dobson, James B. Grand

**Affiliations:** ^1^ Alabama Cooperative Fish and Wildlife Research Unit School of Forestry and Wildlife Sciences Auburn University Auburn AL USA; ^2^ Department of Fish, Wildlife, and Conservation Biology & Graduate Degree Program in Ecology Colorado State University Fort Collins CO USA; ^3^ Department of Mathematics and Statistics Auburn University Auburn AL USA; ^4^ Department of Biological Sciences Auburn University Auburn AL USA; ^5^ U.S. Geological Survey Alabama Cooperative Fish and Wildlife Research Unit Auburn AL USA; ^6^ Present address: Department of Biological Sciences University of Alberta Edmonton AB Canada; ^7^ Present address: U.S. Geological Survey Patuxent Wildlife Research Center Laurel MD USA

**Keywords:** Alaska, Arctic Russia, Bering sea, full annual cycle, integrated population models, Somateria fischeri, spectacled eiders

## Abstract

The Arctic is undergoing rapid and accelerating change in response to global warming, altering biodiversity patterns, and ecosystem function across the region. For Arctic endemic species, our understanding of the consequences of such change remains limited. Spectacled eiders (*Somateria fischeri*), a large Arctic sea duck, use remote regions in the Bering Sea, Arctic Russia, and Alaska throughout the annual cycle making it difficult to conduct comprehensive surveys or demographic studies. Listed as Threatened under the U.S. Endangered Species Act, understanding the species response to climate change is critical for effective conservation policy and planning. Here, we developed an integrated population model to describe spectacled eider population dynamics using capture–mark–recapture, breeding population survey, nest survey, and environmental data collected between 1992 and 2014. Our intent was to estimate abundance, population growth, and demographic rates, and quantify how changes in the environment influenced population dynamics. Abundance of spectacled eiders breeding in western Alaska has increased since listing in 1993 and responded more strongly to annual variation in first‐year survival than adult survival or productivity. We found both adult survival and nest success were highest in years following intermediate sea ice conditions during the wintering period, and both demographic rates declined when sea ice conditions were above or below average. In recent years, sea ice extent has reached new record lows and has remained below average throughout the winter for multiple years in a row. Sea ice persistence is expected to further decline in the Bering Sea. Our results indicate spectacled eiders may be vulnerable to climate change and the increasingly variable sea ice conditions throughout their wintering range with potentially deleterious effects on population dynamics. Importantly, we identified that different demographic rates responded similarly to changes in sea ice conditions, emphasizing the need for integrated analyses to understand population dynamics.

## Graphical abstract

Our understanding of the consequences of climate change for Arctic endemic species is limited. We developed an integrated population model to describe the population dynamics of spectacled eiders, a threatened Arctic sea duck. Our analysis revealed abundance has increased since listing under the Endangered Species Act in 1993, however, spectacled eiders are vulnerable to climate change and the increasingly variable sea ice conditions experienced throughout their core wintering range.

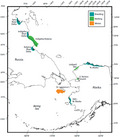

## 1 INTRODUCTION

The Arctic is undergoing rapid and accelerating change in response to global warming. Changes in the abiotic environment have considerably altered biodiversity patterns and ecosystem function across the region (Eamer et al., 2013). Drastic and unprecedented changes in biotic and abiotic processes have had strong and complex impacts on Arctic species including changes in distribution, abundance, extinction risk, and trophic interactions (Macias‐Fauria & Post, 2018). Yet, population responses to climate change remain a critical knowledge gap for many Arctic species (Laidre et al., 2015; Macias‐Fauria & Post, 2018).

While environmental conditions in the Arctic are changing and reshaping ecosystems, the degree to which these changes affect the population dynamics of individual Arctic species is dependent upon their life‐history strategies. Adaptation to rapid climate change is likely to be difficult for long‐lived and highly specialized species making them particularly vulnerable (Berteaux et al., 2004). Frequently, life‐histories of long‐lived organisms have evolved to buffer some demographic rates from environmental variation (Koons et al., 2014; Pfister, 1998; Saether & Bakke, 2000). For instance, in long‐lived species, adult survival is often resilient to stochastic annual variations in environmental conditions. However, interannual variation and directional changes in environmental conditions that exceed historical bounds may influence these demographic rates with important consequences for population dynamics and extinction risk (Schmutz, 2009).

A vast majority of Arctic avifauna are migratory and use the region seasonally to breed, taking advantage of the short but strong burst of resources available in the summer months. Demographic rates respond to spatially and temporally variable environmental conditions, thus requiring detailed information on a species’ spatial distribution throughout the annual cycle to understand those relationships. Processes acting upon individuals may cause immediate or lagged responses in demographic rates across seasons, creating carry‐over or cross‐seasonal effects (Norris & Taylor, 2006; Sedinger & Alisauskas, 2014). Responses may also vary by age or sex or may differ across demographic rates (Rushing et al., 2017). Understanding changes in environmental conditions throughout the annual cycle and their effects on demographic rates is therefore critical for predicting species’ responses to climate change. However, a large portion of what we understand to be climate effects on Arctic avifauna is related to conditions just prior to and during the breeding season (e.g., snow melt dates, phenological mismatches with weather conditions or food resources), or with conditions experienced in temperate or tropical latitudes during the nonbreeding season (Deinet et al., 2015; Fox & Leafloor, 2018; Ganter & Gaston, 2013; Smith et al., 2020). Currently, our understanding of these effects on avian species endemic to the Arctic is extremely limited (Ganter & Gaston, 2013). This limitation is often due to a lack of demographic and spatially relevant environmental data (but see e.g., Sedinger et al., 2011).

Waterfowl are among the most abundant avifauna on Arctic coastal and tundra habitats during the breeding period (CAFF, 2013). Though most species leave the Arctic following the breeding season, most sea ducks (Tribe: Mergini), and more specifically eiders (*Somateria and Polysticta* genera), remain in the Arctic throughout the annual cycle (Ganter & Gaston, 2013; Savard et al., 2015). Spectacled eiders (*Somateria fischeri*) are an endemic Arctic species and can be found in the Pacific Arctic (Bering and Chukchi Seas), Alaska, and Russia, a region experiencing drastic ecosystem change in response to climate change (Huntington et al., 2020). A large sea duck, spectacled eiders spend most of the annual cycle in high latitude coastal and open‐ocean marine habitats and are often associated with sea ice (Flint et al., 2016; Savard et al., 2015; Sexson et al., 2016). Their nonbreeding range remained largely unknown until 1995 when surveys identified large flocks in openings in the sea ice in the mid‐Bering sea (Petersen et al., 1999). The global population of spectacled eiders winters near St. Lawrence Island and migrates to breeding grounds in the coastal tundra wetlands of Alaska and Russia (Sexson et al., 2014). Following marked declines in the western Alaska breeding population the species was listed as threatened under the Endangered Species Act (ESA, as amended 1973) in 1993 (U.S. Fish & Wildlife Service, 1993).

Spectacled eiders use remote areas throughout the annual cycle making it particularly challenging to conduct comprehensive surveys or studies of demography. Several studies have focused on independent analyses of data sets to model population dynamics and demography of spectacled eiders (e.g., Christie et al., 2018; Dunham et al., 2021; Flint et al., 2016); however, estimating abundance and demographic rates has remained challenging. Evidence suggests that all four eider species demonstrate demographic responses, changes in abundance, and distribution shifts related to climate change and environmental conditions encountered throughout the annual cycle (e.g., Barry, 1968; Christie et al., 2018; Fournier & Hines, 1994; Frost et al., 2013; Sexson et al., 2016; Žydelis et al., 2006). Variation in adult survival, breeding propensity, spring arrival dates, clutch size, annual nest success, and duckling survival of closely related common eiders (*S*. *mollissima*) has been linked to environmental conditions experienced during both breeding and nonbreeding seasons (Coulson, 1984; Savard et al., 2015; Waltho & Coulson, 2015). Recent studies suggest shifts in the Bering sea ecosystem have affected adult survival rates and the molting distribution of spectacled eiders (Christie et al., 2018; Flint et al., 2016; Sexson et al., 2016); however, relatively little is known about the effects of changes in environmental conditions on recruitment or population dynamics.

Sea ice is a critical component of spectacled eider wintering habitat (Eamer et al., 2013; Macias‐Fauria & Post, 2018). The Bering sea is dominated by seasonal pack ice, densely packed ice that may drift in response to currents, wind, and storms. Pack ice in the mid‐Bering sea, where spectacled eiders winter, is dynamic and highly variable within and across years (Wang & Overland, 2015). Polynya and open leads in sea ice benefit spectacled eiders offering access to benthic prey and providing roosting habitat (Bump & Lovvorn, 2004; Christie et al., 2018). Concentrated sea ice can dampen the effects of waves from frequent major storms which results in good roosting habitat (Cooper et al., 2013). While extensive ice cover may restrict spectacled eider access to benthic prey and optimal foraging areas (Lovvorn et al., 2003), by contrast, declining sea ice cover has been linked to declines in abundance and shifting distributions of benthic communities, including the preferred prey species of spectacled eiders (Grebmeier, 2012; Grebmeier et al., 2018; Lovvorn et al., 2009, 2015). Sea ice conditions in the Bering Sea are expected to become increasingly variable and sparse (Huntington et al., 2020; Wang & Overland, 2015) with deleterious effects on the habitat for spectacled eiders and other species that are dependent on this marine ecosystem (Smith et al., 2019).

To better understand the effects of climatic change on spectacled eider demography and population dynamics, we developed an integrated population model of the full annual cycle that combined multiple sources of data collected from the western Alaskan breeding population. Here, we use the integrated population modeling framework to (1) estimate demographic parameters (age‐specific survival rates, breeding probability, and productivity), (2) evaluate which demographic rates contribute most to annual population growth, and (3) investigate the effect of environmental conditions on demographic rates throughout the annual cycle. Recently, Christie et al. (2018) documented a nonlinear relationship between sea ice conditions and adult female survival. We were interested in repeating and extending this analysis within an integrated framework. Thus, we evaluate the hypothesis that adult survival will vary in response to extreme sea ice conditions experienced during the wintering period. Similar to Christie et al. (2018), we predicted that adult female survival would be highest in years with intermediate sea ice conditions and decline in years with extremely high or low sea ice conditions, because of restricted access to food versus reduced roosting habitat, respectively. Because the spatial distribution of first‐year birds is generally unknown, we examined the effect of the Arctic oscillation (AO), an important indicator of regional conditions, on first‐year survival to account for general environmental conditions experienced throughout the full annual cycle. Predation pressure and weather conditions (e.g., precipitation) can have strong impacts on annual nest success (DeGregorio et al., 2016; Flint et al., 2016; Mallory, 2015). Therefore, we predicted that annual nest success would decline in years with poor weather conditions (e.g., high precipitation) and in years with high predation rates (e.g., increased fox presence). Furthermore, Lovvorn et al. (2014) documented reduced body condition of spectacled eiders during winter in years with extensive sea ice cover thus, we predicted that annual nest success would be highest in years with average sea ice conditions and decline in years following extreme sea ice conditions in a pattern similar to adult survival. The underlying mechanism for this could involve incubation constancy, whereby females with higher body condition take fewer and shorter recesses from incubation (Blums et al., 1997; Gloutney & Clark, 1991), in turn providing fewer cues to predators and visual exposure of their eggs, respectively. The results presented here provide useful insight for conservation and policy planning regarding the species’ current and future conditions related to climate change.

## 2 MATERIAL AND METHODS

### 2.1 Study area and species

**FIGURE 1:**
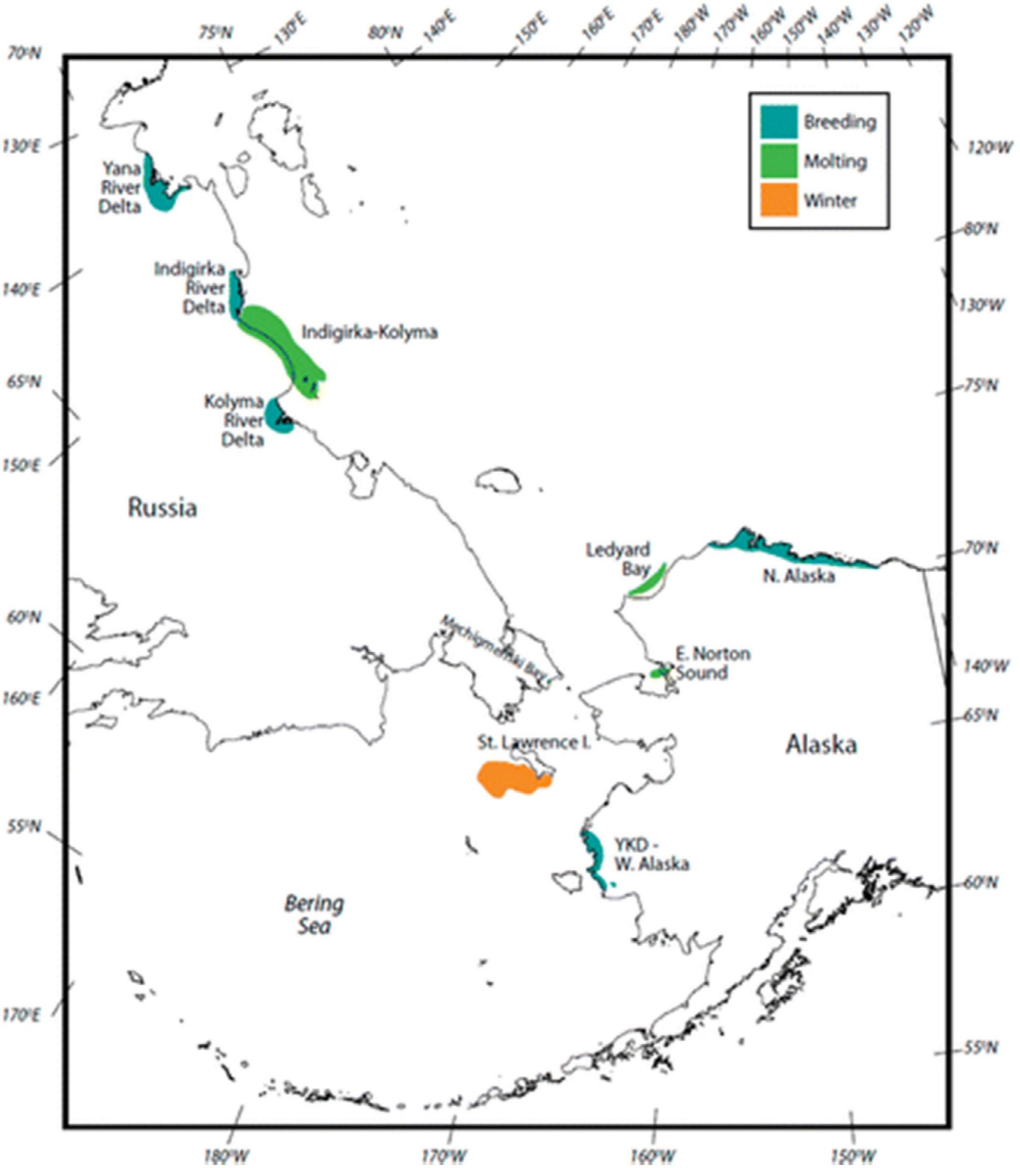
Range map of spectacled eiders *Somateria fischeri* illustrating the three primary breeding areas (Yukon‐Kuskokwim Delta, Arctic Coastal Plain, and Arctic Russia), molting, and wintering areas. (Figure 1 in Flint et al., 2016)

There are two subpopulations of Alaskan breeding spectacled eiders, one population on the Yukon‐Kuskokwim Delta (YKD) and the other on the Arctic Coastal Plain (ACP). Another much larger subpopulation breeds in Arctic Russia (Flint et al., 2016; U.S. Fish & Wildlife Service, 1993) (see map; Figure 1). Both Alaskan subpopulations have been monitored annually since the 1980s using aerial surveys and/or nest monitoring and capture–mark–recapture methods. We focused on the Yukon‐Kuskokwim Delta breeding population, given the existence of long‐term demographic data sets.

The coastal plain of the YKD is one of the largest and most productive waterfowl breeding areas in North America. The YKD is predominately flat tundra and wetlands interspersed with small ponds, lakes, rivers, and tidal sloughs. Spectacled eiders typically arrive on the breeding grounds in late May; males depart 1–2 weeks after incubation begins, and females and their young leave for the wintering grounds in late August. Studies have identified strong breeding and molting site fidelity, important geographical locations, and broad spatiotemporal patterns of spectacled eider site use throughout the annual cycle (Lovvorn et al., 2014; Petersen et al., 1999; Sexson et al., 2014, 2016). The global population of spectacled eiders winters in one distinct region in the Bering Sea south of St. Lawrence Island (Petersen et al., 1999; Sexson et al., 2014). Females marked on the YKD used Norton Sound as their primary staging area during fall. Following the wintering period, individuals previously marked on the YKD either staged along the coast of the Chukotka Peninsula, in Norton Sound, or on the YKD prior to the breeding period (Sexson et al., 2014).

### 2.2 Data collection

Aerial surveys of spectacled eiders have been conducted on 12,832 km^2^ of YKD tundra wetland habitat annually since 1988 (Fischer et al., 2017; Lewis et al., 2019; Platte & Stehn, 2015). Ground‐based surveys have been conducted annually on the YKD since 1985 to estimate the numbers of nests for geese and eiders. These ground‐based surveys sample randomly selected plots within the core nesting area of spectacled eiders in the central coast zone, encompassing 716 km^2^ (Fischer et al., 2017). Eider density varies widely across the YKD with low densities throughout most of the region. Lewis et al. (2019) identified three density‐specific strata: low‐density (0–1.60 nest/km), medium‐density (1.60–3.50 nests/km), and high‐density (>3.50 nests/km). Estimates of nests and aerial observations among low, medium, and high‐density strata on the YKD were used to calculate density‐specific aerial visibility correction factors (VCF) to account for incomplete detection on the aerial surveys. The average density‐specific visibility correction factors were used to convert indices of eider abundance to annual estimates of breeding spectacled eiders and variance (Lewis et al., 2019). Both the estimates of average number of breeding pairs and the annual variance are included as data in the count sub‐model (described below).

On the YKD, survival and productivity studies were carried out on Kigigak Island (60°50’N, 165°50’W) between 1992 and 2015 following protocols established by Grand and Flint (1997; see also Flint et al., 2016). At Kigigak Island, nest searches began in late May and continued through mid‐June. Adult females were captured on nests and given stainless steel leg bands, numbered plastic leg bands, and nasal disks. In some years, brood hens were marked with radio transmitters and monitored to estimate duckling survival (0 – 30 days). At approximately 30 days posthatch, ducklings were captured and marked with stainless steel and plastic bands. Individuals may be marked as 30‐day‐old ducklings or as breeding adults on the breeding grounds. Most marked birds were adults and thus classified as breeding age. Immature and nonbreeding 2‐year‐olds do not come to the breeding grounds and are thus unobservable. Only birds marked as ducklings were of known age upon recapture.

### 2.3 Integrated population model

We developed an integrated population model (IPM) to describe spectacled eider breeding population dynamics for the YKD using annual estimates of abundance from aerial surveys of the entire YKD breeding population and demographic data collected from Kigigak Island from 1992 to 2014. The IPM unified the analysis of aerial survey data on breeding abundance (includes males and females), capture–mark–recapture data (CMR; females only), and productivity data including clutch size at hatch, nest success (1 or more ducklings hatched), and a constant duckling survival rate. Aerial survey data include all relevant information to describe change in abundance over time. CMR data were used to inform age‐specific survival and breeding propensity of 2‐year‐old females. Productivity data were used to inform recruitment of female spectacled eiders into the breeding population.

**FIGURE 2:**
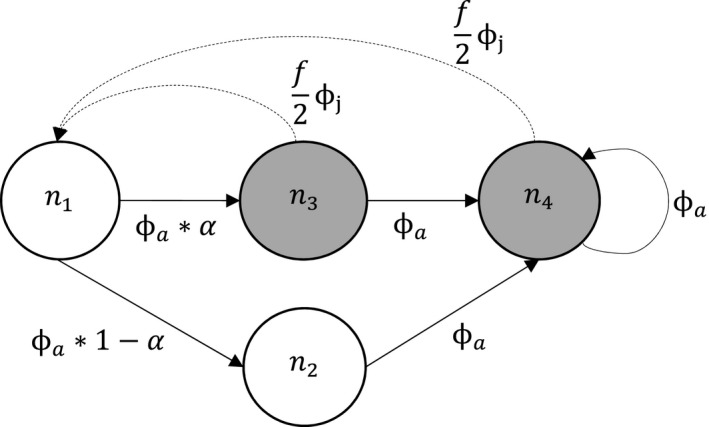
Life cycle diagram of spectacled eiders corresponding to a prebreeding survey female‐only model. Circles represent states, *n*_1_ refers to 1‐year‐olds, *n*_2_ *n*B refers to nonbreeding 2‐year‐olds, *n*_2_B refers to breeding 2‐year‐olds, and *n*_3_+B refers to breeding adult birds 3 years and older. Shaded circles represent the observable portion of the population. Solid lines represent survival and transition probabilities, and dashed lines refer to the recruitment process

We constructed the following matrix projection model based on a prebreeding survey with four stages (Figure 2). To link abundance to demographic rates, we created a projection matrix (**A**) comprised of demographic rates and a vector of stage‐specific abundance *n*_t_:

$${\left[\begin{array}{c} n_{1}\\ n_{2}\\ n_{3}\\ n_{4} \end{array}\right]}_{t+1}=\left[\begin{array}{cccc} 0 & 0 & f_{t}/2*\phi_{t,j} & f_{t}/2*\phi_{t,j}\\ \phi_{t,a}*1‐\alpha & 0 & 0 & 0\\ \phi_{t,a}*\alpha & 0 & 0 & 0\\ 0 & \phi_{t,a} & \phi_{t,a} & \phi_{t,a} \end{array}\right]*{\left[\begin{array}{c} n_{1}\\ n_{2}\\ n_{3}\\ n_{4} \end{array}\right]}_{t}$$

where *n*_1_ is the number of 1‐year‐old females, *n*_2_ is the number of nonbreeding 2‐year‐old females, *n*_3_ is the number of breeding 2‐year‐old females, and *n*_4_ is the number of 3+‐year‐old females, *ϕ*_t,j_ represents annual survival probabilities for first‐year birds, and *ϕ*_t,a_ represents annual survival for birds 1‐year and older (hereafter, adults), and *f*_t_ is the number of fledglings produced per breeding female, divided by 2 because the projection matrix only considers females and the sex ratio is expected to be equal at this life stage (Flint et al., 2016). *t* refers to time, in our case a particular year. We assumed birds 1 year and older had the same survival probability regardless of breeding status because nonbreeding birds (1‐year‐olds and nonbreeding 2‐year‐olds) are unobservable and lack data to inform independent estimates of survival. Female spectacled eiders may begin breeding at 2 years of age, but evidence suggests they are less likely to breed than birds age 3 and older (Flint et al., 2016). Here, we assume all females aged 3 and older breed each year, that is, breeding propensity of adult females is assumed to be 100% (Flint et al., 2016). Direct estimates of 2‐year‐old breeding propensity (α) were unavailable for this species, and thus, we wanted to estimate this demographic rate. This matrix model allows 1‐year‐old individuals to transition to either nonbreeding 2‐year‐old birds with probability (*ϕ*_t,a_ * 1 − α) or breeding 2‐year‐old birds with probability (*ϕ*_t,a_ * α) with corresponding fecundity estimates. We used an annual random effects framework to model survival probabilities, nest success, and recapture probabilities to allow the values to vary over time. However, due to limited data we chose to model breeding propensity of 2‐year‐old females as constant.

### 2.4 Count model likelihood

We used a state‐space model formulation to model the population count data using equations that describe how abundance changes over time. An observation model links the observed population count (index of breeding abundance, including males and females) with the estimated abundance from the state process model. Because our demographic data were specific to female spectacled eiders, our matrix projection model estimated total female abundance, calculated as *N*tot_t_ = (*n*_1_,t + *n*_2_,t + *n*_3_,t + *n*_4_,t). However, our count data were an estimate of breeding abundance which included paired males and females. The number of breeding birds observed during aerial surveys is calculated as a function of the number of breeding pairs and lone males which are assumed to represent a breeding pair (Fischer et al., 2018; Lewis et al., 2019). To link our count data to the overall population model, we calculated the breeding abundance as *N*bpop_t_ = (*n*_3_,t + *n*_4_,t) * 2, assuming an equal sex ratio.

The state process model describes the dynamics of the total population, but our counts only included the breeding males and females. Thus, the observation model linked the observed number of breeding birds (denoted by *y*) to *N*bpop_t_ through the following equation.

*Y*_t_ ∼ Normal (*N*bpop_t_, σ_y,t_).

where σ_y_ is the estimated annual observation error from the aerial surveys and was provided as data. In 2011, aerial surveys were not flown on the YKD, and therefore, there were no observations for that year. We calculated annual changes in abundance (λ_t_ = *N*_t+1_/*N*_t_) and geometric average population growth as derived parameters for the time series. We used weakly informative priors to inform the initial population state with a discrete uniform distribution (Table 1). The complete likelihood for the population count data was *L*_ss_ (*y*, σ^2^_y_ | *ϕ*_a_, α, *N*, *f*).

**TABLE 1:** Parameters, their definitions, and prior distributions used in the spectacled eider integrated population model. Prior distributions were generated based on empirical data for spectacled eiders or other sea duck species

Parameter	Definition	Prior
Capture–recapture and productivity model parameters		
***ɸ*_a_**	Survival of females 1 year and above	Beta (5.5, 1.833) mu =0.75, sd =0.15
***ɸ*_j_**	Survival of first‐year birds (30 days to 1 year)	Beta (2.5, 5.833) mu =0.30, sd=0.15
***α***	Breeding probability of 2‐year‐old females	Beta (2.5, 5.833) mu =0.30, sd=0.15
***p***	Recapture probability of breeding females	Beta (5.056, 5.056) mu =0.5, sd =0.15
***ns***	Nest success (probability of 1 egg hatching)	Beta (5.922, 3.189) mu =0.65, sd=0.15
***cs***	Average clutch size at hatch	Gamma (0.1, 0.1)
***ds***	Survival of ducklings (0 to 30 days)	0.67
***f***	Fecundity ‐ number of ducklings per female $fec_{t}=ns_{t}*cs_{t}*ds$	‐‐
**σ*_ɸ_*_,α,ns_**	Standard deviation of temporal variability	Uniform (0.001, 5)
***ε***	Annual random deviation from the average value $\varepsilon_{t}∼Normal\left(0,\sigma_{\theta }\right)$	‐‐
***β***	Regression coefficients	Normal (0, 10)
Count model parameters		
***n_1_***	Number of immature (1‐year‐old) females $n_{1,t+1}=\left(\frac{fec_{t}}{2}*\phi_{0,t}\right)*\left(n_{3,t}+n_{4,t}\right)$	Discrete Uniform (300, 900)
***n_2_***	Number of 2‐year‐old nonbreeding females $n_{2,t+1}=\phi_{1,t}*\left(1‐\alpha_{t}\right)*n_{1,t}$	Discrete Uniform (10, 200)
***n_3_***	Number of 2‐year‐old breeding females $n_{3,t+1}=\phi_{1,t}*\alpha_{t}*n_{1,t}$	Discrete Uniform (10, 100)
***n_4_***	Number of 3+‐year‐old females $n_{4,t+1}=\phi_{2,t}*\left(n_{2,t}+n_{3,t}+n_{4,t}\right)$	Discrete Uniform (500, 2000)
***Ntot***	Total female abundance $Ntot_{t}=\left(n_{1,t}+n_{2,t}+n_{3,t}+n_{4,t}\right)*2$	‐‐
***Nbpop***	Breeding abundance (males and females) $Nbpop_{t}=\left(n_{3,t}+n_{4,t}\right)*2$	‐‐
***y***	Annual index of breeding abundance	‐‐
***σ_y_***	Annual estimated observation error of *y*	‐‐

### 2.5 Capture‐recapture likelihood

We estimated survival and breeding propensity using capture‐mark‐recapture data from female spectacled eiders banded as ducklings or breeders (2 years or older). During the breeding season between 1992 and 2014, 591 female ducklings (73 recaptures total, 19 recaptured as 2‐year‐old breeders) and 661 adult females (1,335 recaptures) were banded. We used the multistate formulation of the Cormack‐Jolly‐Seber model with unobservable states (Arnason, 1972; Kendall & Nichols, 2002; Lebreton et al., 1992). To decrease computation time and increase efficiency, we converted capture histories into an m‐array and used the multinomial likelihood formulation of the model (Kéry & Schaub, 2012).

We defined a hierarchical model to estimate survival rate in the capture–mark–recapture model (*ϕ*) with a mean and temporal random effect following the general structure:

$$\log it\left(\phi_{t}\right)=\mu_{\phi }+\varepsilon_{\phi ,t}$$

where *ϕ*_t_ is the annual estimate of age‐specific survival probability, *μ*_ϕ_ is the age‐specific mean logit survival, and ε*_ϕ,t_* is the temporal random effect for each age class. Information on the prior distributions for each parameter is available in Table 1.

Recapture probability was conditional on breeding, thus, only breeding birds could be recaptured. We assumed that the probability of detection would be equal for 2‐year‐old and adult breeding females. We modeled recapture probability with a mean and annual temporal random effect:

$$\log it\left(p_{t}\right)=\mu_{p}+\varepsilon_{p,t}$$

where *p*_t_ is the annual recapture probability, *μ*_p_ overall mean logit recapture, and ε*_p,t_* the annual random effect on the logit scale. We assumed the random effect term followed a Normal distribution: ε*_p,t_* ~ Normal(0, $\sigma_{p}^{2}$).

The likelihood of the multistate model was denoted as $L_{\mathrm{CR}}\left(m|\phi_{j},\phi_{a},\alpha ,p\right)$ where ***m*** represents the capture–recapture data which contains information about age‐specific survival (*ϕ*_j_, *ϕ*_a_), breeding propensity (*α*), and recapture probabilities (*p*).

### 2.6 Productivity likelihood

We modeled productivity (*f*) as the product of clutch size at hatch (*cs*), the probability of nest success (*ns*), and duckling survival (*ds*; 0–30 days posthatch). Nests were monitored near the expected hatch date and the number of eggs hatched was recorded to account for egg mortality. Annual clutch size was modeled using a Poisson distribution where the shape parameter was the average clutch size over the time series. Probability of nest success was modeled as proportion of nests with at least one egg hatched out of the total nests recorded. Nest success was modeled using a binomial regression with annual random effects. Duckling survival was supplied as a constant value based on the mean of duckling survival from Kigigak Island (Flint et al., 2016). The likelihood for productivity was $L_{\mathrm{PR}}\left(\mathrm{ns},\mathrm{cs},\mathrm{ds}|f\right)$.

### 2.7 Joint likelihood for the integrated model

The joint likelihood of the integrated population model was the product of the three likelihoods described above and is written as:

$L_{\mathrm{IPM}}\left(m,\mathrm{ns},\mathrm{cs},\mathrm{ds},y,\sigma_{y}^{2}|\phi_{j},\phi_{a},p,\alpha ,N,f\right)=L_{\mathrm{SS}}\left(y,\sigma_{y}^{2}|\phi_{j},\phi_{a},\alpha ,N,f\right)*L_{\mathrm{PR}}\left(\mathrm{ns},\mathrm{cs},\mathrm{ds}|f\right)*L_{\mathrm{CR}}\left(m|\phi_{j},\phi_{a},\alpha ,p\right)$.

### 2.8 Environmental covariates

The descriptions of the model likelihoods and parameters above describe a model that accounted for temporal variation in demographic rates but did not include covariates. Here, we describe the environmental factors across the full annual cycle that we expected to be important influences on demographic change and how they were included in the final model structure. We were particularly interested in understanding how deviations from average sea ice conditions during the “wintering period” (defined as November 1 – April 30) would affect demographic rates. We predicted that nest success and survival of adult females would be affected by sea ice conditions during the wintering period, with lower survival and nest success in years with extreme high or low sea ice cover for reasons described in our introduction. Though breeding propensity of 2‐year‐olds might also be influenced by winter sea ice conditions, we did not have sufficient data to allow breeding propensity to vary over time. The Arctic Oscillation is an index used to describe the pattern of variation in winter sea‐level atmospheric pressure that has been correlated with changes in Arctic climate (Aanes et al., 2002; Thompson & Wallace, 1998). The Arctic Oscillation has also been related to patterns in sea ice thickness and persistence as well as regional weather (Rigor et al., 2002). Thus, it may be a good indicator of habitat conditions spectacled eiders experience throughout the annual cycle.

We calculated the number of days with >95% ice cover (extreme ice days) within the core wintering area during the wintering period as an index of sea ice severity. The core wintering area was identified based on utilization distributions of satellite‐tagged individuals from 1993–1997 and 2008–2012 and confirmed by aerial surveys of the wintering area (Petersen et al., 1999; Sexson et al., 2014). Observed sea ice concentrations were extracted from the core area that spans four grid cells (25‐km resolution) derived from passive microwave satellite imagery using the Bootstrap Algorithm and provided by the National Snow and Ice Data Center. For comparison, we calculated the number of days with <15% ice cover as a metric of extreme low sea ice conditions (Christie et al., 2018). However, we found that extreme ice days and extreme low sea ice conditions are highly correlated (*r* = −0.84, Pearson's correlation coefficient). Given this strong relationship, we chose to include only the standardized number of days with >95% ice cover (hereafter; ice days) and interpret negative deviations from the mean to be representative of low sea ice cover. We included a quadratic effect of “ice days” for adult survival and nest success and modeled the effects using linear models on the logit link scale. Though hatch‐year birds are expected to use the same wintering areas and are thus subject to similar conditions, we expected that due to inexperience, survival would be sensitive to environmental conditions throughout the annual cycle. Thus, we included a linear term for the effect of the Arctic Oscillation on first‐year survival using a linear model on the logit link scale (NOAA, 2019).

Finally, to determine effects of breeding site conditions on nest success we included standardized precipitation during the breeding period (June‐August) as well as an index of fox presence (Fischer et al., 2017). The YKD can experience intense storms along the coast that may cause flooding and total nest failure. Precipitation data were recorded at Bethel Airport in Bethel, Alaska at the National Weather Service Cooperative Network station (Western Regional Climate Center, 2019). Bethel, AK is 108 miles east of Kigigak Island; however, it is the closest weather station. Both arctic foxes (*Vulpes lagopus*) and red foxes (*Vulpes vulpes*) can be found on the YKD and are known nest predators of waterfowl in the region. During nest plot surveys on the YKD, the proportion of nests plots with recent fox sign (e.g., observed fox, scat, fur, tracks, and/or active dens) is recorded annually and we used this as an index of fox presence on the breeding grounds (refer to Table 5 in Fischer et al., 2017). In addition to the quadratic term for extreme ice days, we also included linear terms for precipitation and fox presence in the model for nest success and used a linear model on the logit scale. Prior distributions for all parameters including the regression coefficients are described in Table 2. All covariates were z‐standardized with mean = 0 and standard deviation = 1.

**TABLE 2:** Parameter estimates from an integrated population model including environmental covariates for the Yukon‐Kuskokwim Delta breeding population of spectacled eiders. Model was fit to data collected from 1992 to 2014. Demographic parameter estimates are reported as the mean and 95% Bayesian credible intervals (CRI) on the probability scale. Regression coefficients are reported on the logit scale and correspond to the submodel in the integrated population model (IPM). The covariates included within the submodels include “ice days” which is the number of days where sea ice cover is ≥95% in the core wintering area in the Bering Sea, “arctic oscillation” which is annual index of the Arctic Oscillation pattern, “fox” which is proportion of nest plots with signs of fox, and “precipitation” which is the average rain or snowfall measured at Bethel, Alaska between June and the end of August

Parameter	Mean	95% CRI
Demographic parameters		
Adult survival	0.878	(0.827, 0.921)
Juvenile survival	0.290	(0.148, 0.439)
Breeding propensity of 2‐year‐olds	0.359	(0.223, 0.531)
Nest success	0.778	(0.703, 0.845)
Clutch size	4.297	(3.063, 5.819)
Geometric average *λ* breeding population growth	1.075	(1.064, 1.086)
Regression coefficients		
*β* adult survival linear: *ice days*	−0.132	(−0.549, 0.266)
*β* adult survival quadratic: *ice days*	−0.251	(−0.486, −0.015)
*β* juvenile survival linear: *Arctic oscillation*	0.201	(−0.913, 1.326)
*β* nest success linear: *ice days*	−0.026	(−0.446, 0.401)
*β* nest success quadratic: *ice days*	−0.455	(−0.787, −0.147)
*β* nest success linear: *fox*	−0.169	(−0.604, 0.262)
*β* nest success linear: *precipitation*	−0.032	(−0.417, 0.357)

### 2.9 Model implementation

We fit both the temporal variation model and the model including environmental covariates using Markov Chain Monte Carlo (MCMC) simulations in a hierarchical Bayesian framework using JAGS (Plummer, 2003) software (package “jagsUI”, Kellner, 2016) in R (Versions 4.0.1, R Core Team, 2020). We used 3 chains, each with 900,000 iterations, including an 800,000‐burn in. We thinned by 25, yielding 12,000 posterior samples for each parameter. We assessed convergence of each model based on the Gelman and Rubin statistic (R‐hat between 1 and 1.05) for all parameters. Additionally, trace plots were used to visually confirm adequate convergence of the 3 chains.

### 2.10 Postmodel analysis

We derived annual population growth rate for both the total female abundance (*N*_tot_) and the breeding population (*N*_bpop_) by dividing the abundance in t + 1 by the abundance in t:

$$\lambda_{t}=\frac{N_{t+1})}{N_{t}}$$

We report both population growth rates because they can be used for different purposes. Spectacled eiders are monitored as breeding populations, which includes paired males and females of breeding age, and population growth rate of the breeding population is relevant for conservation and policy planning. We were also interested in assessing the relative contribution of each demographic rate to the realized variation in total female population growth rate. Due to delayed breeding, neither nest success nor first‐year survival directly contribute to the change in breeding abundance within that year. Thus, we calculated the correlation coefficient between each demographic rate and total female population growth. We used the full posterior sample and calculated the probability that the correlations were positive *p* (*r* > 0) (Saunders et al., 2018, 2019; Schaub et al., 2015).

We were interested in understanding how much variation in the demographic rates could be explained by the climatic variables that we considered. We fit a temporal variation model and an environmental conditions model (described above) and included terms for residual variance for each time‐varying demographic parameter. This allowed us to compare the total temporal variance to the residual variance once covariates were included. The amount of temporal variance explained by the covariates was calculated as *V* = (*σ* ^2^_total_ – *σ* ^2^) / *σ* ^2^_total_ where *V* is the proportion of temporal variance explained by including the climate variables, *σ* ^2^_total_ is the total residual variance for each demographic parameter estimated by the temporal variation model, and *σ* ^2^ is the residual variance for each demographic parameter estimated by the environmental conditions model (Kéry & Schaub, 2012).

For both the temporal variation model and the environmental effects model, we assessed the goodness of fit (GoF) of the nest success and count models. We calculated the Freeman–Tukey statistic for the nest success model and calculated a Bayesian *p*‐value. To assess goodness of fit of the count model, we calculated a Bayesian *p*‐value based on the chi‐square statistic.

## 3 RESULTS

### 3.1 Abundance, productivity, and survival

We fit the data using two models, the first included random effects with no covariates to calculate total temporal variation in demographic rates. The second included environmental covariates and random effects and is the model we used for inference. The results reported here refer to the second model including the environmental covariates.

**FIGURE 3:**
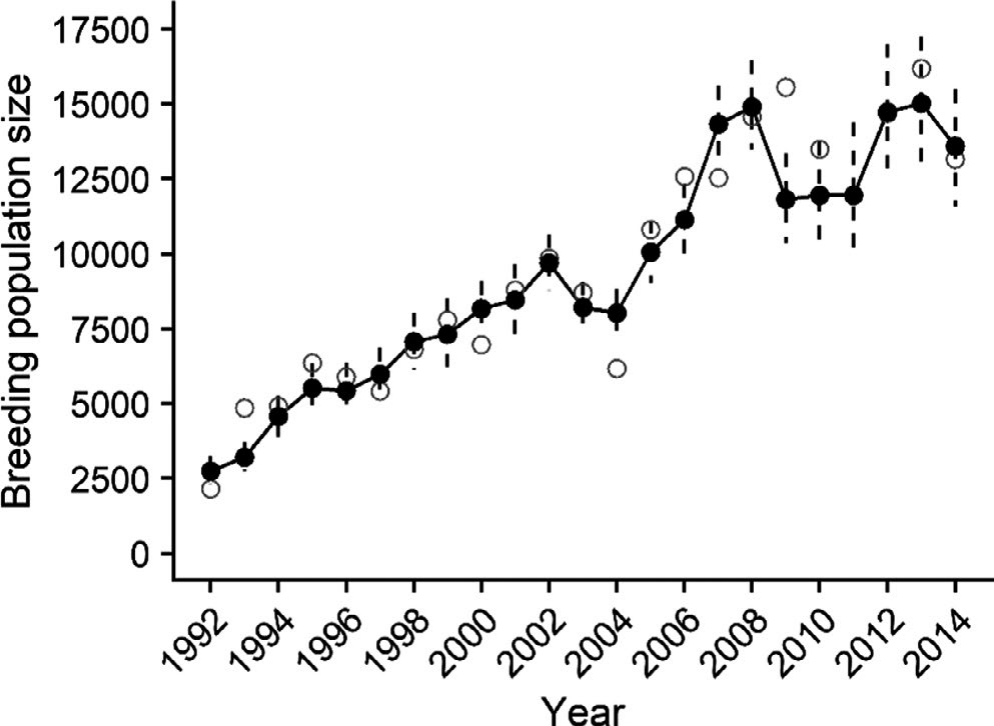
Breeding population size estimates for spectacled eiders breeding on the Yukon‐Kuskokwim Delta in western Alaska. Open gray circles are the point estimates from aerial surveys, black circles, and vertical dashed lines are the annual means and 95% Bayesian credible interval estimates from the integrated population model. Breeding population size includes breeding age males and females

Estimates of abundance and trend indicate that the YKD breeding population has increased over the 23‐year study period (1992–2014; Table 2, Figure 3). Annual population growth rates were variable but mean geometric population growth for the breeding population was 1.075 (95% CRI 1.064, 1.086) indicating an overall positive trend.

**FIGURE 4:**
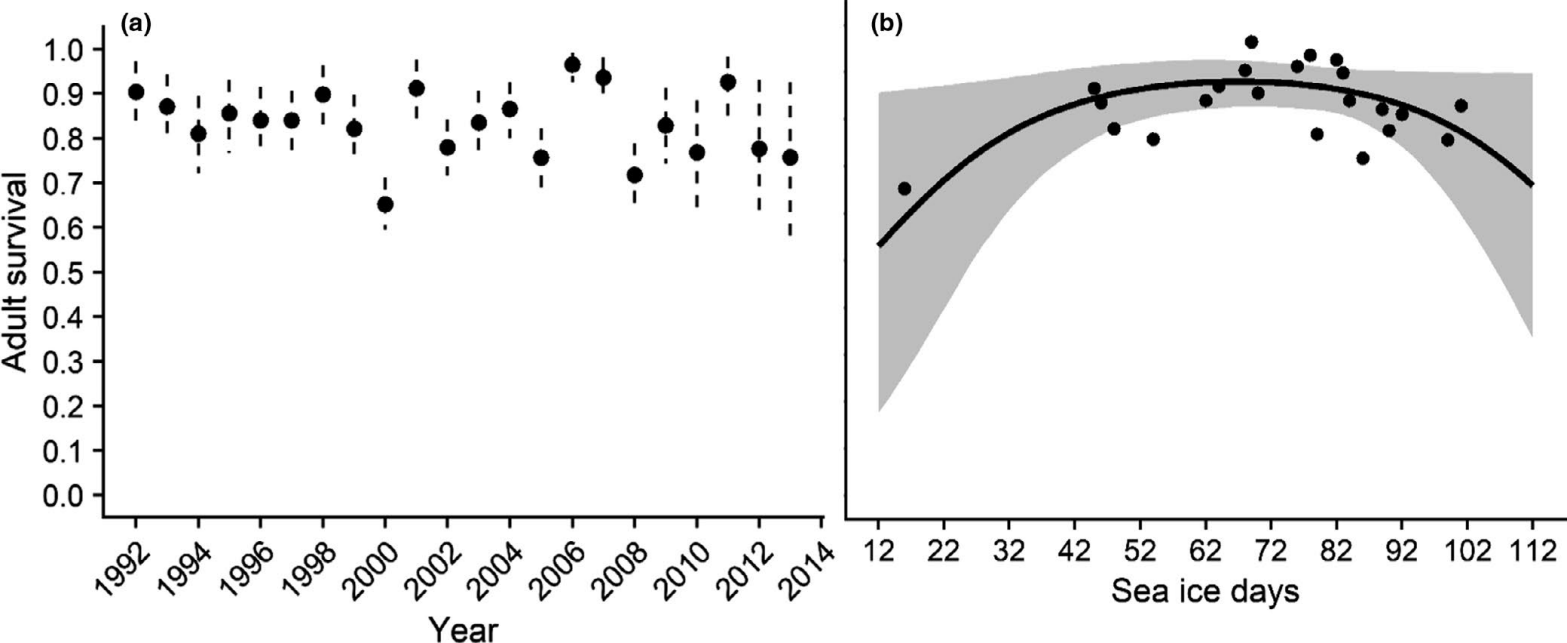
Estimates of annual adult survival of female spectacled eiders (age 1+) breeding on the Yukon‐Kuskokwim Delta in western Alaska (a) and response curves showing the effect of extreme sea ice days (number of days with sea ice concentrations >95%) over the core wintering area on survival of adult female spectacled eiders (b). (a) Black circles and dashed vertical lines are the annual means and 95% Bayesian credible interval estimates from the integrated population model. Annual estimates were generally high and became more variable in the last decade. (b) Black line and gray band are the mean and 95% Bayesian credible interval of the response curve and black circles are the posterior mean estimates of adult survival from the integrated population model. Adult survival is highest in years with intermediate sea ice conditions (50–90 days with extreme sea ice concentrations) and declines with more extreme ice conditions

**FIGURE 5:**
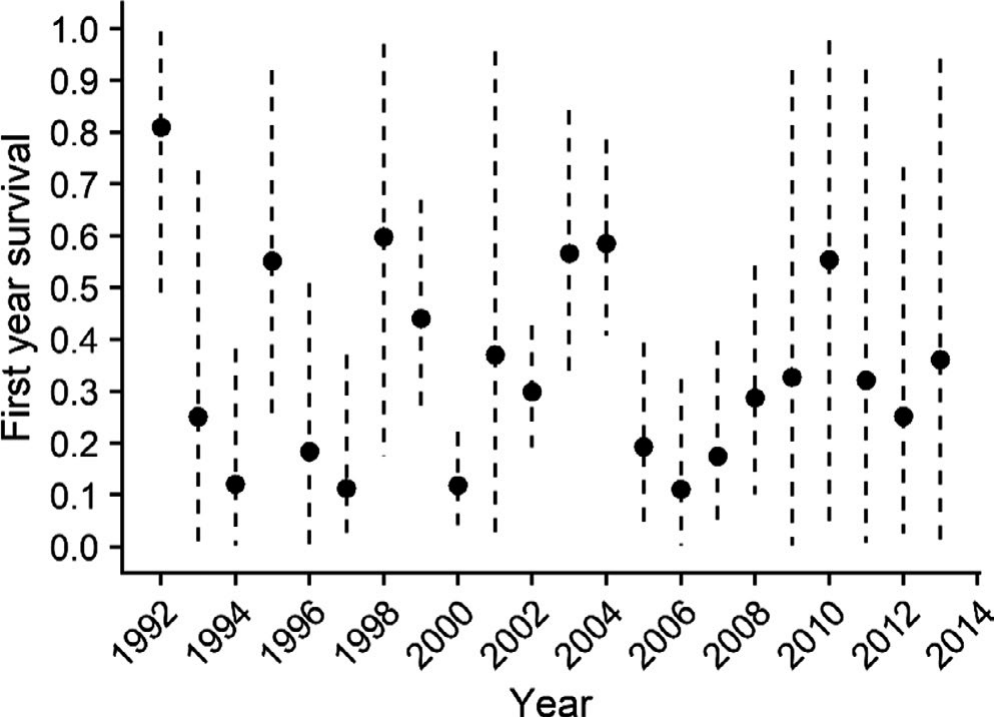
Estimates first‐year survival of spectacled eiders in the Yukon‐Kuskokwim Delta breeding population in western Alaska. Black circles dashed vertical lines are the annual means and 95% Bayesian credible interval estimates from the integrated population model including environmental covariates

**FIGURE 6:**
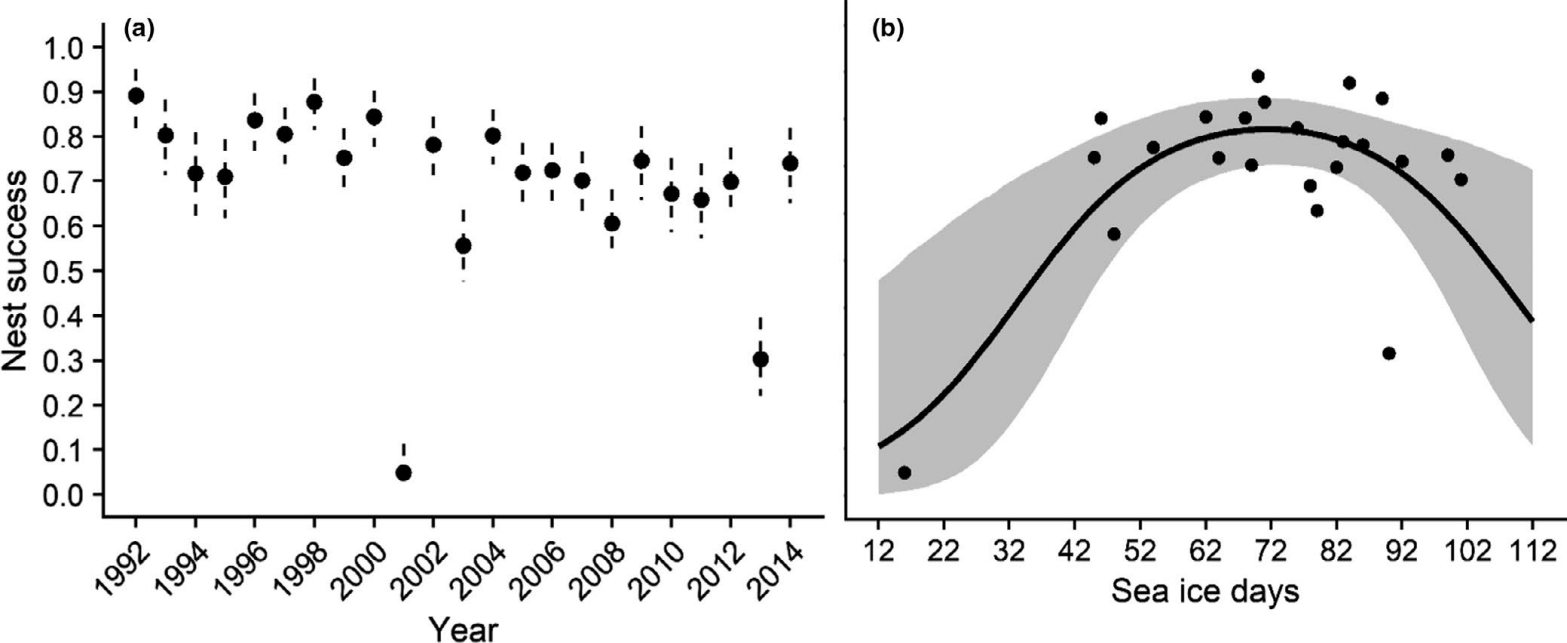
Estimates of annual nest success of female spectacled eiders (age 1+) breeding on the Yukon‐Kuskokwim Delta in western Alaska (a) and response curves showing the effect of extreme sea ice days (number of days with sea ice concentrations >95%) over the core wintering area on nest success of female spectacled eiders (b). (a) Black circles and dashed vertical lines are the annual means and 95% Bayesian credible interval estimates from the integrated population model. Annual estimates were highly variables across time (b) black line and gray band are the mean and 95% Bayesian credible interval of the response curve and black circles are the posterior mean estimates of nest success from the integrated population model. Nest success is highest in years with intermediate sea ice conditions (60–80 days with extreme sea ice concentrations) and declines with more extreme ice conditions

**FIGURE 7:**
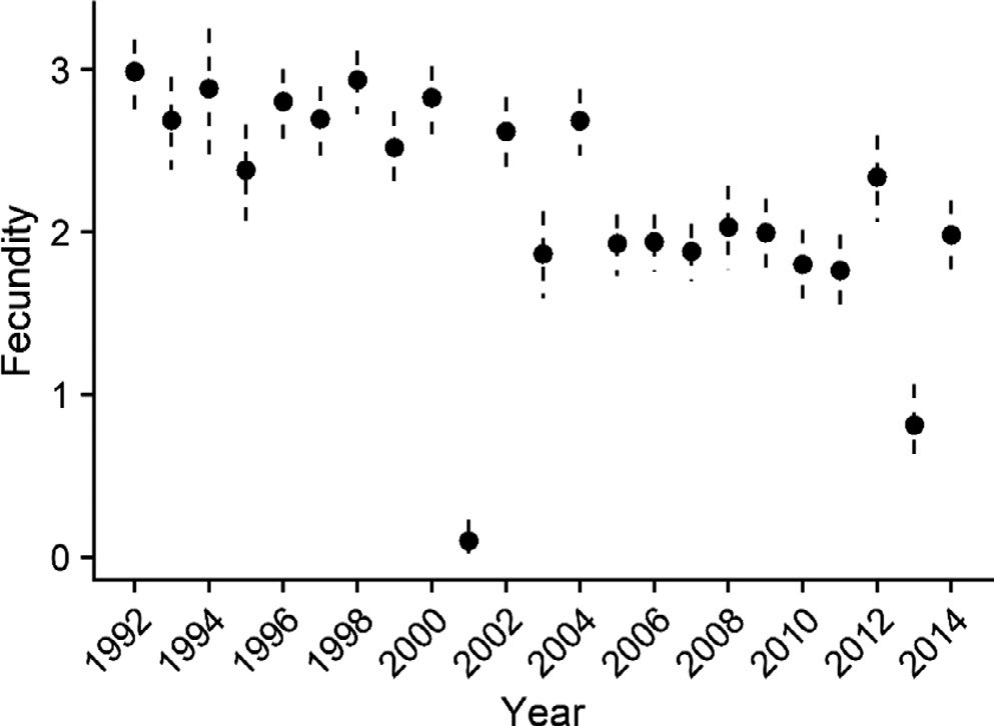
Estimates of fecundity of adult female spectacled eiders in the Yukon‐Kuskokwim Delta breeding population in western Alaska. Fecundity was estimated as the product of nest success, clutch size at hatch, and duckling survival (included as a constant) and estimates the expected number of female ducklings that survive to 30 days posthatch. Most of the annual variation in fecundity is a function of the variation in nest success, thus, the two parameters are highly correlated. Black circles dashed vertical lines are the annual means and 95% Bayesian credible interval estimates from the integrated population model including environmental covariates

Mean adult survival rate (age 1+) was high (0.878; 95% CRI 0.827, 0.921), and though adult survival rate was generally stable, annual point estimates were more variable over the past 10 years of the study (Table 2, Figure 4a). Mean apparent first‐year survival rate was 0.290 but annual estimates were variable, and precision was low (Figure 5). Mean breeding propensity of 2‐year‐old females was 0.359, which is consistent with estimates from other eider species (Table 2). Average probability of nest success was 0.778 but highly variable across years with significant declines in 2001 and 2013 (Figure 6a). Average clutch size at hatch was 4.297 with 95% CRI between 3.06 and 5.82 (Table 2). Because fecundity (*f*) was derived from the product of nest success, clutch size, and duckling survival (a constant), the values varied over time primarily in response to changes in nest success (Figure 7) and fecundity and nest success probability were highly correlated (*r* = 0.93, Pearson's correlation coefficient).

### 3.2 Environmental effects on demographic rates

The number of extreme sea ice days (days with ≥95% sea ice cover) on the core wintering area fluctuated between 16 and 101 days over the study period (1992–2014). Only one year (winter 2001) within the study period had a particularly low number of extreme sea ice days (16 days ≥95% ice cover; November 2000 – April 2001) which coincided with the lowest annual estimates of adult survival rate and nest success probability.

Here, we define “support” for an effect of a covariate on a demographic rate if the 95% credible intervals of the posterior on the slope parameters do not cross zero. Based on the posterior distributions of the slope parameters, we found support for a quadratic relationship between the number of extreme sea ice days and adult survival and a similar relationship for annual nest success (Table 2). Adult survival was highest in years when the number of sea ice days was between 50 and 90 (Figure 4b). Nest success was highest when the average number of sea ice days was between 60 and 80 and annual success declined with an increase or decrease in the number of extreme sea ice days (Figure 6b). Based on posterior estimates, we found no support for a relationship between nest success and fox presence or precipitation (Table 2). In addition, we found no evidence of a relationship between first‐year survival and the annual Arctic Oscillation index.

The inclusion of covariates explained 16% of the temporal variation in adult survival (total temporal variance on the logit scale =0.71 (95% CRI: 0.28,1.41), residual temporal variance = 0.59 (95% CRI: 0.20, 1.28), and 44% of the temporal variation in nest success (total temporal variance on the logit scale = 1.13 (95% CRI: 0.55, 2.02), residual temporal variance = 0.64 (95% CRI: 0.28, 1.34). The inclusion of the Arctic Oscillation covariate for first‐year survival did not explain temporal variation. Furthermore, the Bayesian *p*‐values for the nest success model with temporal variation only was 0.298 but was 0.490 when the environmental covariates were included. These values indicated that the addition of covariates improved model fit. Alternatively, the Bayesian *p*‐values for the count model were 0.50, regardless of the inclusion of covariates; thus, both models fit the count data well.

### 3.3 Demographic contributions to population growth

**FIGURE 8:**
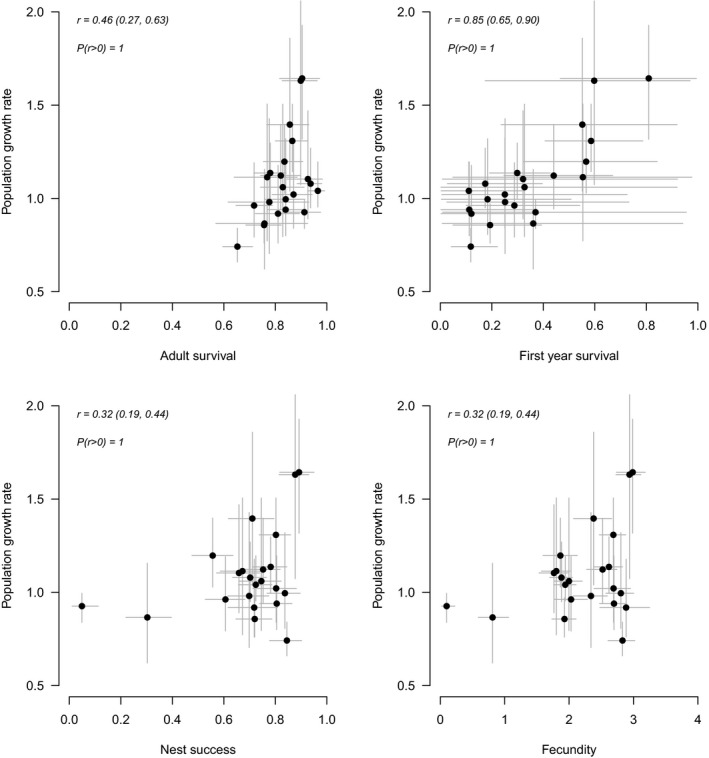
Annual posterior means of population growth rate plotted against annual posterior mean estimates of adult survival (a), first‐year survival (b), annual nest success (c), and fecundity (d). First‐year survival was the demographic rate most strongly correlated with variation in annual population growth. Black dots indicate mean estimates of the demographic rates with corresponding 95% Bayesian credible intervals (gray lines), *r* is the posterior mode of the correlation coefficients with corresponding 95% CRI, and *p*(*r* > 0) indicates whether the correlation was positive

First‐year survival was highly correlated with variation in female population growth rates (*r* = 0.85) and the 95% CRI excluded zero (Figure 8). Adult survival rate was positively correlated with total female population growth *r* = 0.46 (95% CRI 0.27, 0.63) (Figure 8). Both the probability of nest success and fecundity were positively correlated with population growth *r* = 0.32 (95% CRI 0.19, 0.44) though less so than adult or first‐year survival rates.

## 4 DISCUSSION

We investigated the full annual cycle population dynamics of spectacled eiders using an integrated population model and found that the climatic conditions experienced during the wintering period affect both adult survival and nest success (Figures 4b and 6b). Previous research identified a similar relationship between adult survival and winter sea ice (Christie et al., 2018); however, conditions outside of the breeding season were never considered in analyses of reproductive success (Flint et al., 2016). Integrating data on multiple demographic rates provided us with additional information on important demographic processes that influence population dynamics. Specifically, we were able to estimate mean breeding propensity for 2‐year‐old females and annual survival of first‐year birds, quantities that were not estimable using any of the data streams independently. Among the parameters that were allowed to vary over time, we determined that annual variation in female population growth was largely influenced by first‐year survival, demonstrating additional benefits of using an integrated modeling approach.

Extreme winter sea ice conditions contributed to annual variation in both adult survival and nest success. Severe environmental conditions during the nonbreeding period can influence eider body condition via effects on resource availability, access to resources, and energetics (Christie et al., 2018; Cooper et al., 2013; Lovvorn et al., 2009, 2015). The Bering Sea is a highly productive benthic ecosystem; however, warming temperatures and declining sea ice have caused distributional shifts and overall reductions in benthic prey available to spectacled eiders (Grebmeier et al., 2018; Lovvorn et al., 2009). In years with heavy ice cover across the core wintering area, individual body condition was documented to be poor in response to restricted openings in the ice and subsequent lack of access to suitable prey (Cooper et al., 2013; Lovvorn et al., 2014). Alternatively, sea ice may dampen the impact of waves and provide roosting areas for individuals during the nonforaging period, thus significantly reducing thermoregulation costs (Lovvorn et al., 2009). A species’ tolerance to environmental conditions is limited due to physiological and ecological constraints, which accounts for nonlinear relationships between demographic rates and climate (Jenouvrier, 2013). Several studies have identified “bell‐shaped” or otherwise nonlinear relationships between climate covariates, demographic rates, and body condition in sea birds (e.g., Ballerini et al., 2009; Barbraud et al., 2011; Gremillet et al., 2015). These relationships are similar to the results documented between sea ice conditions, adult survival, and nest success (Christie et al., 2018, *this study*).

Severe winter sea ice conditions can reduce body condition (Cooper et al., 2013; Lovvorn et al., 2014) affecting survival during winter and migration to spring staging or breeding grounds. We found that adult survival was highest when the number of ice days was between approximately 50 and 90 days, over the 180‐day wintering period, and subsequently declined beyond those limits. Our results provide further evidence for a nonlinear effect of winter sea ice conditions and survival for ice‐dependent avifauna (Ballerini et al., 2009; Barbraud et al., 2011; Christie et al., 2018; Gremillet et al., 2015; Jenouvrier et al., 2012). Previous analyses of spectacled eider survival between 1992 and 2004 found support for a linear effect of winter sea ice conditions (Flint et al., 2016), with the additional years of data we were able to uncover a more complex pattern between survival and climatic variation. However, the addition of the quadratic terms for ice days accounted for only 16% of the temporal variation in adult female survival, suggesting other factors are also important. For example, female survival may also be affected by reproductive costs, though these may be mediated to some degree. Predation by mammalian predators and ingestion of spent lead shot on the breeding grounds may also contribute to adult female mortality (Flint & Grand, 1997, Grand et al., 1998, Flint et al., 2016) with the latter suspected to be a factor propelling the initial population decline (USFWS 1996). Though we did not explore these sources of mortality, research quantifying the relative effects of different stressors on adult survival is certainly warranted.

Eiders are capital breeders and thus rely on energy stores acquired prior to breeding for sustenance during the incubation period. Females can lose 26% of their body mass during incubation (Flint & Grand, 1999). Arriving at the breeding grounds in poor condition may force females to increase the frequency and duration of incubation recesses to feed, potentially exposing nests to harsh environmental conditions or increased predation risk and resulting in total or partial nest failure (Criscuolo et al., 2002; D'Alba et al., 2010; Iles et al., 2013; Lehikoinen et al., 2006). Furthermore, females in poor body condition may abandon their nests at higher rates, in favor of survival and reproduction in the following year. Nest success is estimated to be highest between 60 and 80 ice days, indicating that nest success may be more sensitive to winter sea ice conditions than adult survival. This is consistent with a life‐history trade‐off between survival and reproductive success for long‐lived iteroparous species such as eiders (Orzack & Tuljapurkar, 2001; Saether & Bakke, 2000).

We linked nest success to environmental conditions during the nonbreeding and breeding seasons. However, we found no evidence for an effect of fox presence or precipitation during the breeding season on annual nest success. It is possible that both our index of fox abundance and precipitation were not accurate metrics of how predation and weather influence spectacled eider nest success. Mammalian predation is a major cause of partial or total nest failure for ground‐nesting species (DeGregorio et al., 2016; Mallory, 2015; Quinlan & Lehnhausen, 1982). Foxes are a major nest predator for waterfowl, however, the data on fox abundance on the Yukon‐Kuskokwim Delta are limited and measured based on the proportion of plots with signs of fox; thus, this index may not adequately capture predator‐prey dynamics. Unfavorable weather conditions during the nesting period were found to have negative effects on nest success and recruitment of common eiders (Iles et al., 2013; Jónsson et al., 2013). However, the closest weather station to Kigigak Island is nearly 180 km east in Bethel, Alaska and storms and precipitation in coastal regions are often more extreme than those experienced further inland. In the Arctic, severe storm frequency is expected to continue to increase with complex regional impacts on precipitation (Terenzi et al., 2014) and potentially deleterious effects on nest success, duckling survival, and nesting habitat (Jorgenson et al., 2018). Furthermore, phenology of waterfowl nesting on the YKD is related to the timing of snowmelt and nest success (Lindberg et al., 1997; Sedinger & Raveling, 1986; but see Babcock et al., 2002). Depending on the timing, coastal storms and subsequent flooding can cause localized nest failure or duckling mortality through a number of mechanisms including drowning, exposure to cold water temperatures, and stunted growth when exposed to salt water prior to developing salt glands (Devink et al., 2005; Grand & Flint, 1997; Iles et al., 2013). Both predation and habitat conditions have been identified as major influences on survival of spectacled eider ducklings (Flint et al., 2006). Efforts to gather data on duckling survival in response to weather conditions, habitat change, and predation during the breeding season may help us understand the relative impacts of changes in recruitment at the population level and identify potential conservation actions.

Estimating survival of first‐year spectacled eiders is a considerable challenge because of delayed breeding and lack of information on geographic distribution of 1‐year‐olds. Inference can only be made based on small sample sizes of birds banded as ducklings and recaptured as breeders (2 years or older). On average, first‐year apparent survival was 0.29, which is slightly lower than rates estimated for other eider species (Koneff et al., 2017) but broadly similar to those of other sea duck species in Alaska (e.g., common goldeneyes; Lawson et al., 2017). This value, however, was generally consistent with estimates for spectacled eiders from other studies (Christie et al., 2018; Flint et al., 2016). Previous analyses did not estimate annual survival of first‐year birds and were thus unable to test for an effect of environmental conditions. Prior attempts to monitor spectacled eiders produced no data on the space use of first‐year birds (Sexson et al., 2014). Because we know so little about the distribution of first‐year birds, we chose to use the Arctic Oscillation as an index for climate conditions that first‐year birds may experience but were unable to detect any relationship. Our results indicate that change in first‐year apparent survival had the strongest correlation with variation in female population growth rates. These results are consistent with those of Saether and Bakke (2000) who found that highly variable demographic rates may contribute more to variation in population growth than the demographic rates that contribute most strongly to asymptotic population growth. Further efforts to gather such information may reduce uncertainty in annual estimates of survival and identify appropriate environmental conditions that may affect survival and ultimately variation in population growth of spectacled eiders.

Estimates of breeding propensity of 2‐year‐old birds are broadly similar to estimates obtained for sea ducks (Koneff et al., 2017). Much like first‐year survival, estimating breeding propensity is challenging because of low samples sizes for females banded as ducklings and returning in their second year to breed. Furthermore, it is not possible to determine the age of birds first marked as breeders, though we would expect that some unknown portion of individuals are initially marked as 2‐year‐olds. It is also likely that breeding propensity of 2‐year‐olds is related to environmental conditions during the nonbreeding season. However, we do not have the data required to make such inference and had to constrain this parameter to be constant over time. In this study, we assumed that breeding propensity of adults (3+ years) was 100% each year. Many females in our data set were recaptured multiple years in a row, which provides support for this assumption. However, intermittent breeding has been identified in other eider species and may be linked to environmental conditions faced throughout the annual cycle (Coulson, 2010; Hanssen et al., 2013; Jónsson et al., 2013; Mehlum, 2012). If spectacled eiders were exhibiting nonbreeding rates similar to common eiders (e.g., up to 70% nonbreeding), we may expect the annual estimates of abundance to vary rather substantially in response (Coulson, 2010). However, this was not the case in our analysis or in analyses of the abundance data alone (Dunham et al. *in review*, Fischer et al., 2018). Further research is warranted to determine whether spectacled eiders demonstrate a trade‐off between survival and reproduction (i.e., bet‐hedging strategy; Saether & Bakke, 2000) through intermittent breeding following severe winter or spring conditions, which may account for some variation in breeding abundances across years.

Breeding abundance increased substantially over our study period (Figure 3), and our abundance estimates and trend were largely consistent with recent analyses of the abundance data alone (Dunham et al., 2021; Fischer et al., 2018). Eiders exhibit a slow “pace of life” strategy, which often includes high variance (“boom and bust”) in annual reproductive success and first‐year survival (Orzack & Tuljapurkar, 2001). In structured populations with delayed breeding, we would expect temporal variation in recruitment to affect population structure and subsequently abundance over the long term (Gaillard et al., 2008; Pfister, 1998). It is likely that intermittent years of high recruitment could have strongly contributed to the overall positive trend in population growth, despite additional variation in adult survival in recent years. For example, in species with delayed recruitment such as spectacled eiders, variation in first‐year survival can contribute greatly to both recruitment and population dynamics, as we found. But because we had to assume constant duckling survival and 2‐year‐old breeding propensity, and 100% breeding propensity thereafter, we did not pursue a more formal retrospective decomposition of female population growth rates (e.g., Koons et al., 2016). Our inference about the relative contribution of demographic parameters to past variation in population growth rates is limited to the parameters that were allowed to vary over time in our integrated population model. Furthermore, sea ice conditions through our study period were often “intermediate” (i.e., within one standard deviation from the mean) and extreme years happened less than half of the time. Therefore, while annual variation in sea ice conditions may affect demographic rates, it is unlikely that the negative effects were severe or frequent enough to cause the population to decline.

Our results add to the evidence that variable sea ice conditions over the wintering period affect spectacled eider demography (Christie et al., 2018; Flint et al., 2016). However, during the study period there was only a single year with sea ice conditions well below average. In 2001, the Bering Sea experienced record low sea ice extent and this year coincided with the lowest estimates of nest success and adult survival. We acknowledge that the extreme value in 2001 has considerable influence on our inference, and the relationship between below‐average sea ice conditions and demographic parameters is uncertain (Christie et al., 2018 and see our Supplementary Material). This uncertainty is partially reflected in the credible intervals of the posterior estimates of the regression coefficients (Table 2), in the predicted response curves showing the effect of extreme ice days on survival and nest success (Figures 4 and 6). To further address the potential influence of the extreme value of “ice days” in 2001, we include a modified model fit without the 2001 covariate values (Table S1). Nevertheless, we note that the low sea ice extent during the winter of 2001 is not a singular anomaly nor the result of a measurement error. Sea ice extent in the Bering Sea has remained well below average for three years in a row (2017–2019), with a new record low set in 2018 (Huntington et al., 2020). Though efforts resumed in 2019, continuous capture–recapture data were only available through 2015 and we were unable to model the effects of an extended number of years with minimal sea ice on spectacled eider demography. The recent observations of declining sea ice conditions combined with evidence of negative effects of such conditions on demography should further motivate detailed demographic studies of spectacled eiders. Studies on individually marked birds in addition to intensive nest monitoring would offer the greatest opportunity to test our findings.

Changes in sea ice persistence may have important effects on spectacled eider diets and range dynamics that compound the deleterious effects predicted by our results. Declines in sea ice concentrations in the Bering Sea are expected to affect the benthic faunal composition and biomass that supports the marine ecosystem and provides food for Arctic marine predators (Grebmeier, 2012; Grebmeier et al., 2006, 2018). Furthermore, projected changes in sea ice will increase the duration and extent of open water periods, likely altering the spatial distribution of suitable refugia and affecting the spatial structure of benthic communities (Zhang et al., 2012). Since the 1990s spectacled eiders have shifted their molting distribution, likely in response to changing ecosystem conditions (Sexson et al., 2016). How spectacled eiders adapt (e.g., prey switching, range shifts) in response to sea ice loss throughout their wintering habitat remains to be seen. Further research on adaptations to changing sea ice conditions will be critical for understanding spectacled eider's short and long‐term responses to climate change.

Understanding Arctic species demographic responses to environmental conditions has become increasingly important in a changing climate. Using the integrated population modeling approach, we were able to identify limiting factors affecting population growth via different demographic rates throughout the annual cycle. We believe this study provides further evidence of the importance of long‐term demographic studies to identify demographic responses to climate change and identify opportunities for conservation action.

## CONFLICT OF INTEREST

The authors indicate there are no competing interests.

## AUTHOR CONTRIBUTION

**Kylee Dunham:** Conceptualization (lead); Data curation (lead); Formal analysis (lead); Methodology (lead); Project administration (supporting); Visualization (lead); Writing‐original draft (lead); Writing‐review & editing (equal). **Anna M Tucker:** Formal analysis (supporting); Methodology (supporting); Visualization (supporting); Writing‐review & editing (equal). **David Koons:** Formal analysis (supporting); Methodology (supporting); Writing‐review & editing (equal). **Ash Abebe:** Conceptualization (supporting); Formal analysis (supporting); Methodology (supporting); Writing‐review & editing (equal). **F. Stephen Dobson:** Conceptualization (supporting); Methodology (supporting); Writing‐review & editing (equal). **James Barry Grand:** Conceptualization (supporting); Formal analysis (supporting); Funding acquisition (lead); Methodology (supporting); Project administration (lead); Resources (lead); Supervision (lead); Writing‐review & editing (equal).

## Supporting information

Supplementary Material

## Data Availability

All data and code used in these analyses are accessible through Dryad. https://doi.org/10.5061/dryad.4qrfj6q88
