# Supplementary material for: Demographic responses to climate change in a threatened Arctic species
Source: Ecol Evol. 2021 Jul 14;11(15):10627–43. doi: 10.1002/ece3.7873 (PMC8328435; doi:10.1002/ece3.7873)
Supplement: Supplementary file 1 — Supplementary Material [file ECE3-11-10627-s001.docx]

**Demographic responses to climate change in a threatened Arctic species**

Kylee D. Dunham, Tucker, A.M., Koons, D.N., Abebe, A., Dobson, F.S., and J.B. Grand.

**Supplementary material**

During the study period (1992-2014) there was only a single year with sea ice conditions well below average. In 2001, the Bering Sea experienced record low sea ice extent and this year coincided with the lowest estimates of nest success and adult survival. In response to reviewer concerns regarding the strength of evidence for the effects of below-average sea ice conditions on spectacled eider adult survival and nest success, we have run an additional model fit without the covariate values for 2001. We report the methodological changes and the results below.

The model, priors, and parameters are all the same as those outlined in the manuscript. We estimated nest success and adult survival in all years, however, did not estimate the effect of covariates on nest success or adult survival in 2001. Both parameters ($\theta$, i.e., nest success and adult survival) for 2001 were estimated using the central mean and Gaussian random effects: ($logit(\theta\left[ 2001 \right])=mean.\theta+ \varepsilon.\theta[2001]$, where $\varepsilon.\theta\left[ 2001 \right]\sim Norm(0, \sigma_{\theta}^{2})$. In all other years (1992-2000, 2002-2014) the demographic parameters were modeled as described in the main text, with the effects of the corresponding environmental covariates and random effects terms. More specifically, we estimated the effects of ‘ice days’, ‘fox presence’, and ‘precipitation’ on demographic parameters between 1992-2000 and 2002-2014. This allowed us to fit the model using all the data, account for the inherently low estimates of survival and nest success in 2001, and estimate the effects of the environmental covariates without the influence of the extreme conditions in 2001. Here, we report the results focusing on the estimated demographic parameters and regression coefficients (Table S1).

Estimates of demographic parameters from this model fit without the covariate values in 2001 were largely the same as those presented in the main text, with overlapping 95% credible intervals between corresponding parameters (see Table S1 here and Table 2 in main manuscript). In the modified data set, the number of extreme sea ice days (days with ≥ 95% sea ice cover) on the core wintering area fluctuated between 45 and 101 days over the study period. As in the main manuscript, covariates that were ‘supported’ were defined as covariates with 95% credible intervals on slope parameters that did not include zero. Based on the posterior distributions from the modified model we found no support for a relationship between environmental covariates and demographic parameters (Table S1). Though we note that the credible interval for the quadratic term of ‘ice days’ narrowly missed our criteria with 89.9% of the posterior indicating a negative value, which is consistent with results presented in the main text.

We ran this supplementary analysis in response to reviewer concerns about fitting our model and making inference about the effects of low sea ice conditions with large influence from the record low value in 2001. Importantly, we identified a slightly less precise but still biologically important effect of extreme winter sea ice conditions on adult survival. However, we did not detect a similar effect of sea ice days on nest success. Of note, both adult survival and nest success were lowest in 2001 regardless of whether environmental covariates were included or not. We further emphasize that the record low sea ice conditions experienced in the Bering Sea in 2001 were not the result of a mistake in data collection or measurement error, nor was it a singular anomaly. Since 2014, the winter sea ice conditions in the Bering Sea have been consistently below average with a new record low for winter sea ice set in 2018. However, intensive capture-mark-recapture surveys ended in 2015 and thus we are unable to model the effects of consistently low sea ice on spectacled eider demography. Sea ice concentration in the region is expected to continue declining with increased variability in seasonal ice conditions (Wang and Overland 2015). Demographic rates may be affected differently by the frequency, duration, and amplitude of extreme events (Jenouvrier 2013). The results of the collective analyses serve to highlight the need to monitor dynamic systems over long time periods to capture the effects of extreme conditions on demography (Clutton-Brock and Sheldon 2010).

Literature Cited

Clutton-Brock, T., and B.C. Sheldon. 2010. Individuals and populations: the role of long-term, individual-based studies of animals in ecology and evolutionary biology. Trends Ecol Evol (10):562-73. doi: 10.1016/j.tree.2010.08.002.

Jenouvrier, S. 2013. Impacts of climate change on avian populations. Global Change Biology 19: 2036-2057. doi: 10.1111/gcb.12195.

Wang, M., and J.E. Overland. 2015. Projected future duration of the sea-ice-free season in the Alaskan Arctic. Progress in Oceanography 136: 50-59.

| Table S1. Parameter estimates from a modified integrated population model of the Yukon-Kuskokwim Delta breeding population of spectacled eiders. Model was fit to demographic data collected from 1992 to 2014. However, this model only estimates the effects of environmental covariates on demographic parameters from 1992-2000 and 2002-2014 to avoid quantifying the effect of extreme sea ice declines in 2001 per the reviewer’s request. Demographic parameter estimates are reported as the mean and 95% Bayesian credible intervals (CRI) on the probability scale. Regression coefficients are reported on the logit scale and correspond to the sub model (i.e., survival or nest success) and parameter (e.g., adult survival or juvenile survival) in the integrated population model (IPM). The covariates included within the sub models include ‘ice days’ which is the number of days where sea ice cover is ≥95% in the core wintering area in the Bering Sea, ‘arctic oscillation’ which is annual index of the Arctic Oscillation pattern, ‘fox’ which is proportion of nest plots with signs of fox, and ‘precipitation’ which is the average rain or snowfall measured at Bethel, Alaska between June and the end of August. | | |
| --- | --- | --- |
| Parameter | Mean | 95% CRI |
| **Demographic Parameters** |  |  |
| Adult survival | 0.877 | (0.816, 0.933) |
| Juvenile survival | 0.288 | (0.141, 0.442) |
| Breeding propensity of 2-year-olds | 0.363 | (0.229, 0.533) |
| Nest success | 0.674 | (0.530, 0.794) |
| Clutch size | 4.305 | (3.096, 5.812) |
| **Regression Coefficients** |  |  |
| $\beta$ adult survival linear: *ice days* | -0.026 | (-0.525, 0.514) |
| $\beta$ adult survival quadratic: *ice days* | -0.358 | (-0.988, 0.207) |
| $\beta$ juvenile survival linear: *Arctic oscillation* | 0.21 | (-0.947, 1.41) |
| $\beta$ nest success linear: *ice days* | -0.196 | (-0.857, 0.436) |
| $\beta$ nest success quadratic: *ice days* | 0.214 | (-0.538, 0.947) |
| $\beta$ nest success linear: *fox* | -0.354 | (-0.993, 0.264) |
| $\beta$ nest success linear: *precipitation* | -0.057 | (-0.623, 0.514) |
